# Wet Sheets in Diarrhœa

**Published:** 1871-12

**Authors:** 


Wet Sheets in Diarrhœa.—Oppenheimer employed this treatment in twenty cases of rapid diarrhœa in children, from fourteen days to four years old, with three deaths and seventeen reçoveries. In two cases of chronic diarrhœa there was no result. He used sheets wet in water at 50° to 55° Fahr., in which the little sufferer was enveloped and then covered with a blanket, which was kept on until perspiration set in—say from half an hour to an hour. Internally the patients were given ice-water, broth, barley-water, milk, wine or brandy, but no medicine. As soon as perspiration occurred, the packing was removed, the child wiped dry, and cloths dipped in cold water laid upon its abdomen. and renewed as fast as they grew warm. Where the diarrhœa persisted, the packing was repeated within an hour. If cerebral congestion occurred, the packing was discontinued and ice applied to the head. The treatment seems suited only to acute attacks of diarrhœa in children.—*Schmidt's Jahrbucher.*

